# Exploring the diversity of endophytic fungi and screening for their pullulanase-producing capabilities

**DOI:** 10.1186/s43141-021-00208-0

**Published:** 2021-07-29

**Authors:** Bindu Naik, S. K. Goyal, Abhishek Dutt Tripathi, Vijay Kumar

**Affiliations:** 1 Department of Agricultural Engineering (Formely Farm Engineering), Institute of Agricultural Sciences, Banaras Hindu University, Varanasi, UP 221005 India; 2 Centre of Food Science and Technology, Institute of Agricultural Sciences, Banaras Hindu University, Varanasi, UP 221005 India; 3 Department of Biosciences, Swami Rama Himalayan University, Swami Rama Nagar, Jolly grant, Dehradun, Uttarakhand 248140 India

**Keywords:** Pullulanase, Endophytes, Fungi, *Aspergillus*, *Penicillium*, Bindu Naik is the first author of the manuscript.

## Abstract

**Background:**

Pullulanases are the significant industrial group in the 13 glycosyl hydrolases category, known as the α-amylases family. There are very few reports on pullulanase from fungal sources. Based on the above research gap, the present study was undertaken to explore the endophytic fungi for their pullulanase-producing capabilities.

**Results:**

A total of 126 endophytes were isolated from *Tradescantia pallida*, Zea *mays*, and *Trifolium alexandrinum. Aspergillus*, *Penicillium*, and *Ganoderma* species recovered highest from the stem of *Tradescantia palida*. *Fusarium* was dominant in the stem and leaf of *Zea mays. Penicillium*, *Aspergillus*, *Ganoderma*, *Cladosporium*, *Fusarium*, and *Alternaria* were recovered from the *Trifolium alexandrium.* The Shannon index in *Tradescantia pallida* was highest in leaves while in *Zea mays* and *Trifolium alexandrinum*, it is highest in the stem. The Simpson’s index is highest in the case of *Zea mays* stem and root. Species richness was indicated by Menhinick’s index, and it was found that this value was highest in the roots of *Trifolium alexandrinum.* As per our knowledge, no comparative data is available on the endophytic diversity of the above plants taken for the study. Out of 126 endophytes, only 2.38% produced pullulanase while 7.94% produced amylase. The recovery of pullulanase-producing endophytic fungi was very less. But the importance of pullulanase is high as compared to amylase because it has both α-1,6 and α-1,4 hydrolyzing ability. Therefore, the most promising isolates were identified by ITS sequence analysis. Based on spore chain morphology, isolates BHU-25 and BHU-30 were identified as *Penicillium* sp. and *Aspergillus* species, respectively. This is the first report of pullulanase from endophytic *Aspergillus* and *Penicillium*.

**Conclusion:**

Endophytes *Aspergillus* sp. and *Penicillium* sp. produce pullulanase enzyme. This is the first report of pullulanase from endophytic *Aspergillus* and *Penicillium*.

**Supplementary Information:**

The online version contains supplementary material available at 10.1186/s43141-021-00208-0.

## Background

Endophytes are microorganisms that are found inside the tissues of the host and perform ecological relationships without causing any harm to the host. They have been distributed throughout nature and are the source of various novel biomolecules such as enzymes, antibiotics, antioxidants, and anticancer compounds [1]. The endophytes also provide resistance to the plants by secreting secondary metabolites [2]. The fungal diversity is associated with different tissues of the same host plant and is also dependent on its geographical distribution and climatic conditions [3]. Nowadays, it is an emerging challenge to analyze the diversity of conglomerated fungal endophytes for the discovery of novel biomolecules producing species and their role in the ecosystem. Hence, the diversity of various endophytic microbes have been explored for their metabolic potential [4–7]. Pullulanase constitutes an important group of industrial enzyme which belongs to a family of 13 glycosyl hydrolases, also called as the α-amylase family [8, 9]. They hydrolyze the glycosidic bonds in the starch during the saccharification process which leads to the production of glucose, maltose, and maltotriose syrups. Pullulanase is produced by animals, plants, fungi, and bacteria. Among bacteria, many mesophilic, thermophilic, and hyperthermophilic bacteria and archaea have been reported to produce pullulanase. This enzyme is distributed mostly among bacteria like *Clostridium* spp*.*, *Bacillus* spp., certain species of *Bacillus*, and *Geobacillus* [10–12]. Bacterial pullulanase has a high production cost and low yield which are major limitations in the industrial production of this enzyme. The production cost of pullulanase can be minimized by selecting agro-industrial waste as the substrate for enzyme production under solid-state fermentation (SSF) processes, which can mainly be achieved by fungi. Various agricultural wastes such as wheat bran, rice bran, corn cobs, soy hull, and sugarcane bagasse have been successfully used for the production of various metabolites especially enzymes in solid-state fermentation [13, 14]. However, very little or limited information is available on fungal pullulanase. In this context, the present study was undertaken to explore the fungal diversity of endophytes associated with *Tradescantia pallida*, *Trifolium alexandrinum*, and *Zea mays* having pullulanase-producing capabilities.

## Methods

The plants were collected from Varanasi, Uttar Pradesh, India, in January 2014. The samples which were used in the experiment and their voucher numbers are given in Table 1. The samples were identified from the Botanical Survey of India, Dehradun, Uttarakhand, India.

**Table 1:** Plant taken during the study for the isolation of endophytic fungi

V.No.	Plant	Plant parts	Place
BHUIAS-2	*Tradescantia pallida*	Root, leaf, stem	Varanasi
BHUIAS-3	*Zea mays*	Root, leaf, stem	Varanasi
BHUIAS-4	*Trifolium alexandrinum*	Root, leaf, stem	Varanasi

### Isolation and screening of fungi-producing pullulan hydrolyzing enzyme

The different plant parts were used for the isolation of endophytic fungi. The parts of the plants were washed with running tap water. The surface sterilization was done according to the method described previously [15]. The plant parts were cut with a sterilized sharp blade into small pieces and plated on potato dextrose agar medium and incubated at 25°C for 4–5 days. The isolates were screened for their ability to hydrolyze the pullulan by the agar plate method. The fungal strains were inoculated on pullulan agar medium and incubated at 25°C for 72 h. The plates were flooded with iodine and observed for the clear zone around the fungal colonies. The solid-state fermentation was carried in 250 ml flask containing 05g (dry weight) powdered solid substrate supplemented with nutrient salts—1% (NH_4_)_2_SO_4_, 1% KH_2_PO_4_, 0.2% NaCl, and 0.2% MgSO_4_. The flasks containing the above media were sterilized (121°C for 20 min.) and cooled, then added 1 mL of spore suspension and incubated at 27°C for 5 days. Overall 60% initial moisture content was maintained in the flask. The agro-based wastes such as wheat bran were obtained from the local market of Varanasi have been used as a solid substrate. After fermentation, the flasks were flooded with phosphate buffer (pH 6.5) and the enzyme was harvested by centrifuging at 10,000g for 10 min. The extract was used as the crude enzyme. Pullulanase activity was assayed by the DNS method [16]. The same extract was used for well diffusion assay on pullulan agar plates (1% pullulan and 1.5% agar). 0.1 mL of the enzyme sample and 0.4 mL phosphate buffer (pH 6.5) were added to 0.5 mL of 1% (w/v) solution of pullulan. The reaction mixture (substrate plus enzyme) was incubated for 30 min at 40°C. Added 1 ml DNS reagent and incubation of test tubes were performed 5–10 min in a boiling water bath. Then, after cooling down, 0.5ml of 1% (w/v) sodium-potassium tartarate solution was also added. The final volume was adjusted to 5ml by adding 2.5ml of sterile distilled water. The absorption was measured at 570 nm afterward. The protein has been estimated by Lowry et al. [17] method.

### Diversity index

The Shannon diversity index (*H*), Simpson’s Diversity Index, and Menhinick’s index were determined according to the method described by Chowdhary and Kaushik [18].

### Identification of the endophytic fungi

The promising isolates were initially identified through colony and spore chain morphology according to the method described previously [19]. For genotypic identification, genomic DNA was isolated from the endophytic fungi by using a genomic DNA isolation kit (Chromous Biotech, Bangalore, India). The endophytic fungi were grown in potato dextrose broth (PDB) and after 48 h centrifuged at 10000 rpm for 10 min to obtain mycelium. This mycelium is used for the preparation of genomic DNA by using DNA extraction kit as per kit instructions (Chromous biotech, Bangalore, India). PCR amplification of the ITS region (650–700bp) of the fungal endophytes was performed using two primers ITS1: TCCGTRSGNGAACYTGHGG and ITS4: TCCTCCGCTTATTKATDTGC. The final reaction mixture of volume 100μL contained PCR buffer F, 1.5mM MgCl2, 200μM of each dNTP, 400ng of each primer, 2.5 U Taq DNA polymerase (Genei, Bangalore, India), and 100 ng template. The amplification was carried out in an Eppendorf Thermo-cycler 96 with the following protocol: a 5-min denaturation stage at 94°C, followed by 35 amplification cycles at 94°C for 30 s, 52°C for 30 s, and 72°C for 45 s, and a 5-min extension step at 72°C. Agarose gel electrophoresis was used for the detection of PCR products and was visualized by ultraviolet (UV) fluorescence after ethidium bromide staining. PCR products were purified by HiPurA^TM^ PCR product purification spin kit (HiMedia Laboratories, Mumbai, India) according to the manufacturer’s instructions. The ABI PRISM® Big Dye® Terminator version 3.1 Cycle Sequencing Kit (Applied Biosystems, Foster City, CA) was used to sequence PCR products according to the manufacturer’s instructions using ITS1 and ITS4 primers. The sequences of selected isolates were also analyzed using the BLAST (Blastn) search engine (http://www.ncbi.nlm.nih.gov) and submitted to the GenBank database (NCBI).

### Molecular characterization of most promising isolates

The software included in the MEGA X package was used for phylogenetic and molecular evaluation [20]. The ITS sequences of the fungal type strains were aligned with the respective type fungal nucleotide sequences obtained from GenBank using the CLUSTAL W [21] program. The maximum likelihood method was used to study evolutionary history by using the Kimura 2-parameter model [22]. The consensus bootstrap tree derived from 1000 replicates represents the evolutionary history of the analyzed species. Branches that match partitions in bootstrap replicates reproduced in 50% collapse [23].

## Results

### Biodiversity of endophytic fungi

**Fig. 1:**
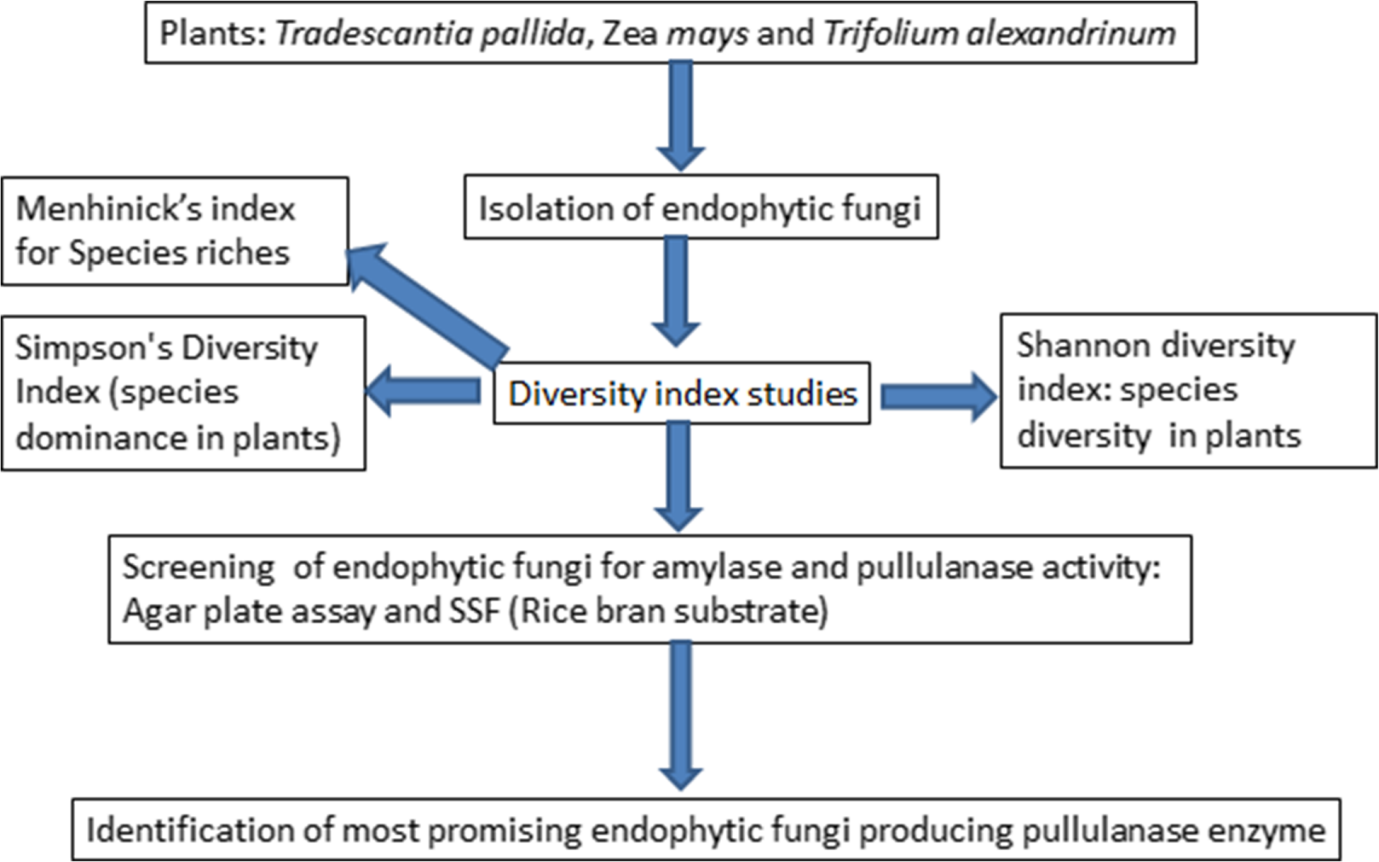
The schematic representation of the present study

**Fig. 2:**
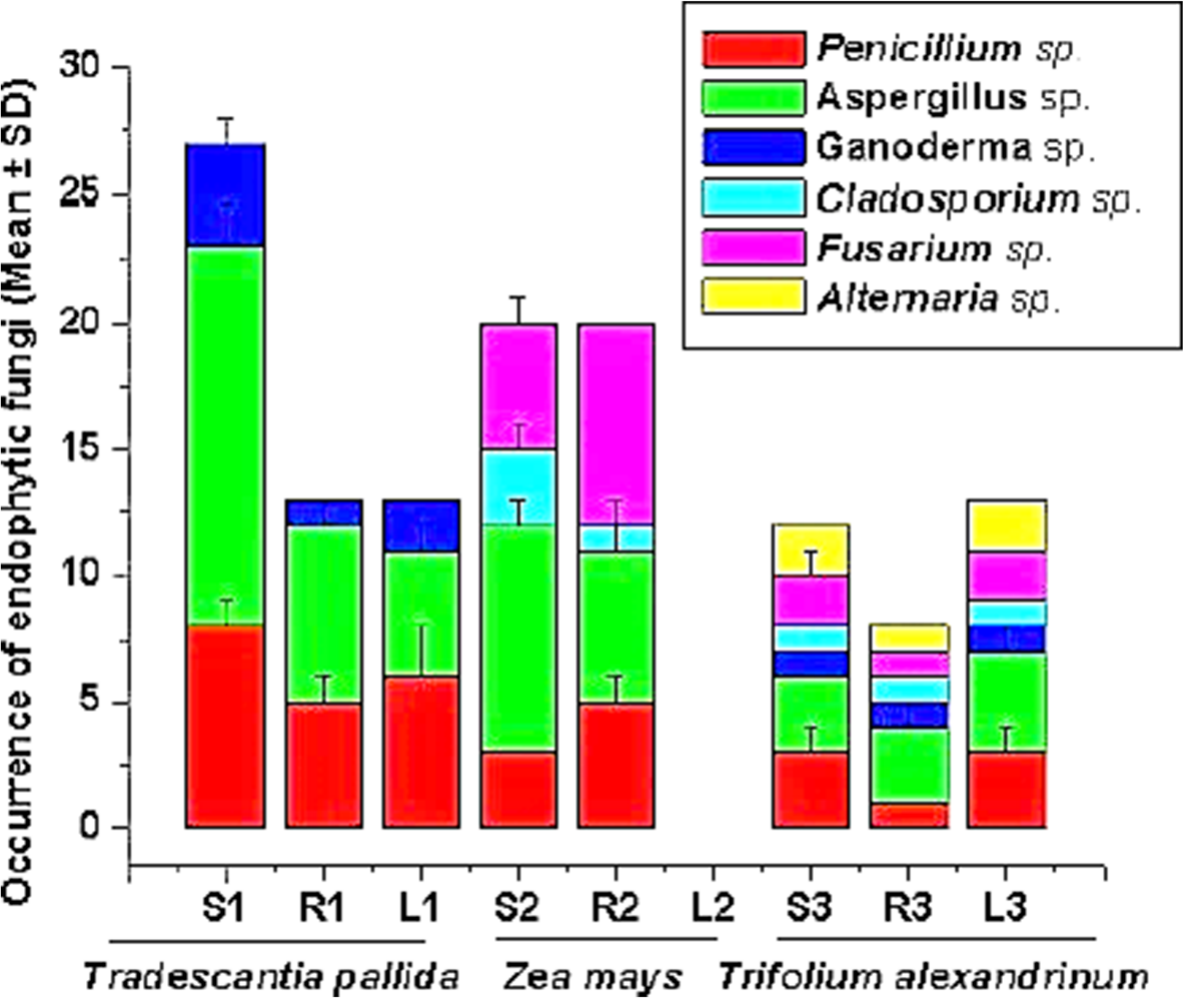
Distribution of endophytic fungi in different parts (stem, root, and leaves) of *Tradescantia pallida* (S1- the stem, R1-root, L1-leaf) Zea *mays* (S2- the stem, R2- root, L2-leaf) and *Trifolium alexandrinum* (S3-stem, R1-root, L3-leaf; bar indicates the ±standard deviation

The schematic representation of the present study has been shown in Fig. 1. A total of 126 endophytes were isolated from plants under study and were identified based on cultural characteristics and spore chain morphology. The distribution of endophytic fungi in different parts (stem, root, and leaves) of *Tradescantia pallida*, Zea *mays*, and *Trifolium alexandrinum* has been given in Fig. 2. *Aspergillus*, *Penicillium*, and *Ganoderma* species recovered highest from the stem of *Tradescantia palida*. *Fusarium* was dominant in the stem and leaf of *Zea mays. Penicillium*, *Aspergillus*, *Ganoderma*, *Cladosporium*, *Fusarium*, and *Alternaria* were recovered from the *Trifolium alexandrium.* This finding suggests that *Aspergillus* is most commonly associated with plant endophytes*.*

The diversity index is a quantitative indicator that measures the number of different species and the degree of distribution of individuals among those species. The Shannon index in *Tradescantia pallida* is highest in leaves, While in *Zea mays* and *Trifolium alexandrinum*, it is highest in the stem. The Simpson’s index is highest in the case of *Zea mays* stem and root as shown in Table 2. Species riches are indicated by Menhinick’s index, and it is found that this value is highest in the roots of *Trifolium alexandrinum.* As per our knowledge, no comparative data is available on the endophytic diversity of the above plants taken for the study.

**Table 2:** Alpha diversity of endophytic fungi

Diversity index	***Tradescantia pallida***			***Zea mays***			***Trifolium alexandrinum***		
	Stem	Root	Leaf	Stem	Root	Leaf	Stem	Root	Leaf
**Total species**	03	03	03	04	04	00	06	06	06
**Total isolates**	27	13	13	20	20	00	12	08	13
**Shannon index**	0.970	0.898	1.012	0.928	0.858	00	0.900	0.888	.898
**Simpson index**	0.582	0.556	0.615	0.977	0.937	00	0.806	0.781	0.793
**Menhinick’s index (Dmn)**	0.577	0.832	0.832	0.894	0.894	00	1.732	2.121	1.664

### Occurrence of endophytic fungi-producing amylase and pullulanase

**Fig. 3:**
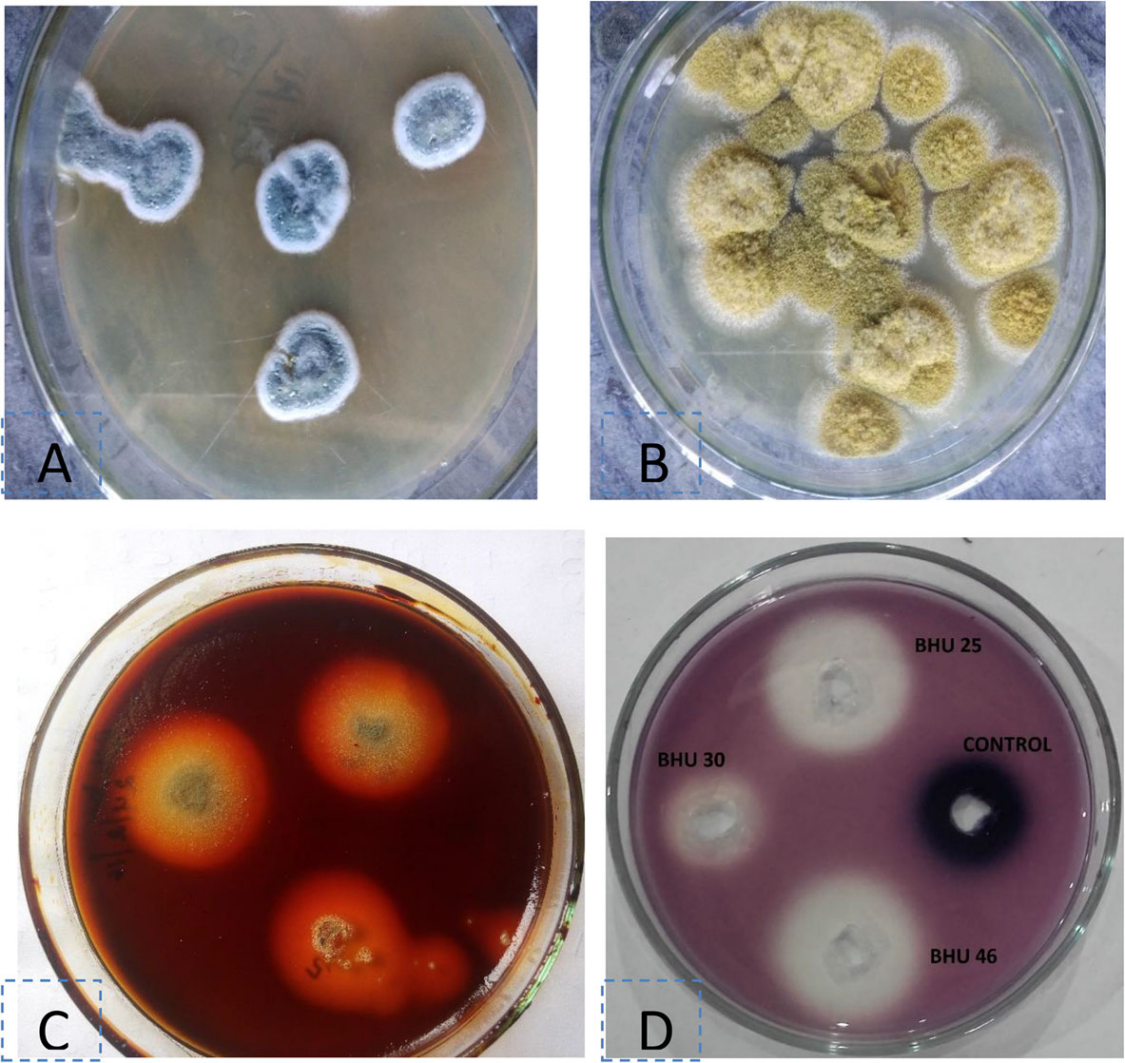
Colony morphology of isolates **a** BHU-25-1; **b** BHU-46, **d** hydrolysis of pullulan by the isolates BHU-25, and BHU-46 plate flooded with iodine

The plates showing the clear zone around the colonies and wells indicate the presence of pullulanase enzyme (Fig. 3c, d and Table S1). Out of 126 endophytes, only 2.38% produced pullulanase while 7.94% produced amylase (Fig. 2). The recovery of pullulanase-producing endophytic fungi was very less. But the importance of pullulanase is high as compared to amylase because it has both α-1,6 and α-1,4 hydrolyzing ability. Therefore, the most promising isolates producing pullulanase were taken for secondary screening (in solid-state fermentation using rice bran as a substrate). In solid-state fermentation using rice bran (agro-waste) as substrate, an enzyme activity of 8.24±1.38 U/gds (protein, 2.1±0.36mg/mL) and 6.14 ±1.03 U/gds (protein 1.8±0.26 mg/mL) has been obtained by the isolates BHU-25 and BHU-46, respectively.

### Characterization of promising isolates

**Fig. 4:**
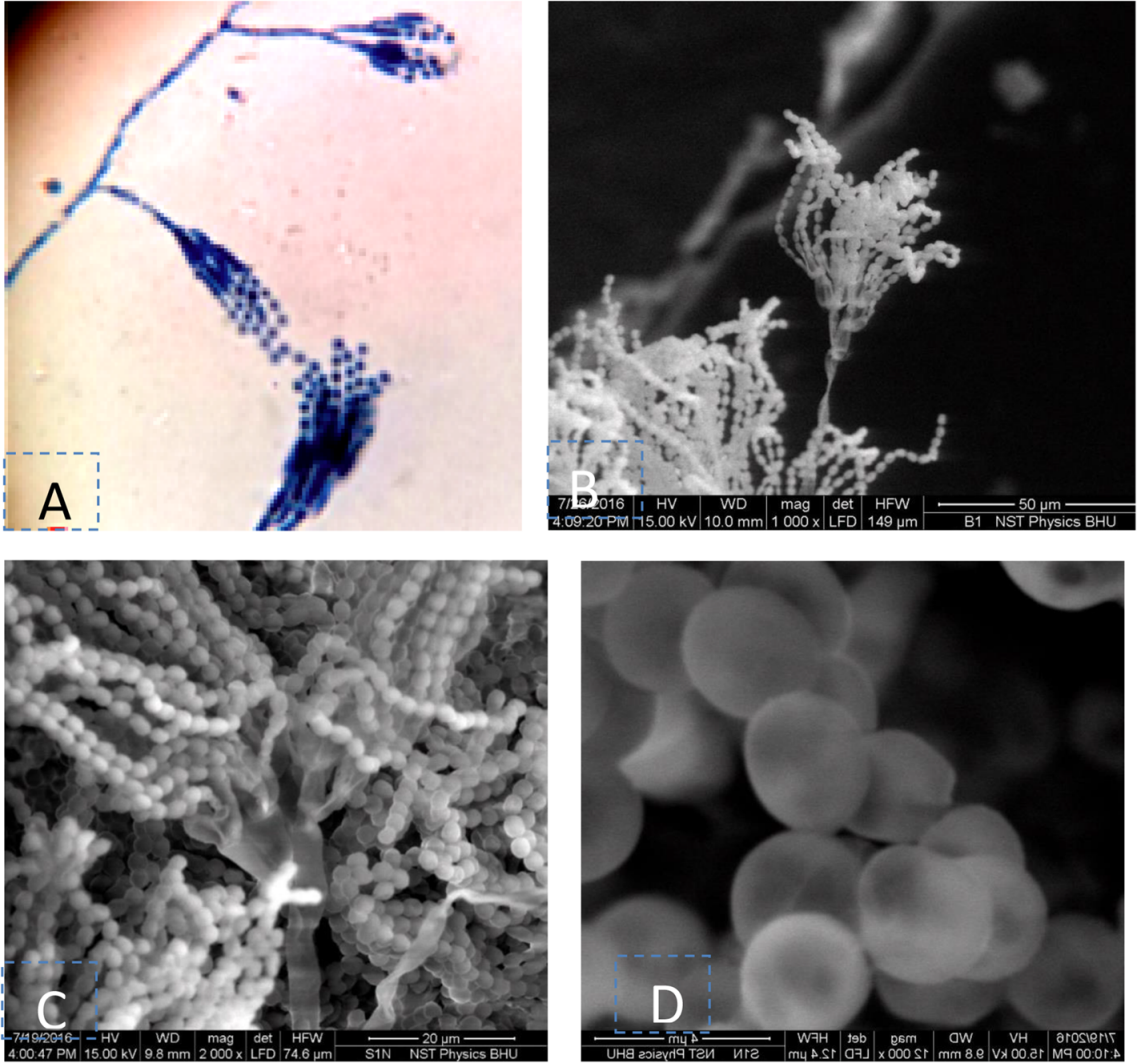
Spore chain morphology of *Penicillium* sp. BN-1, **a**-at 1000X (light microscope); **b**-ESEM at 1000X; **c**-2000X; **d**-Spore surface (12,000X)

**Fig. 5:**
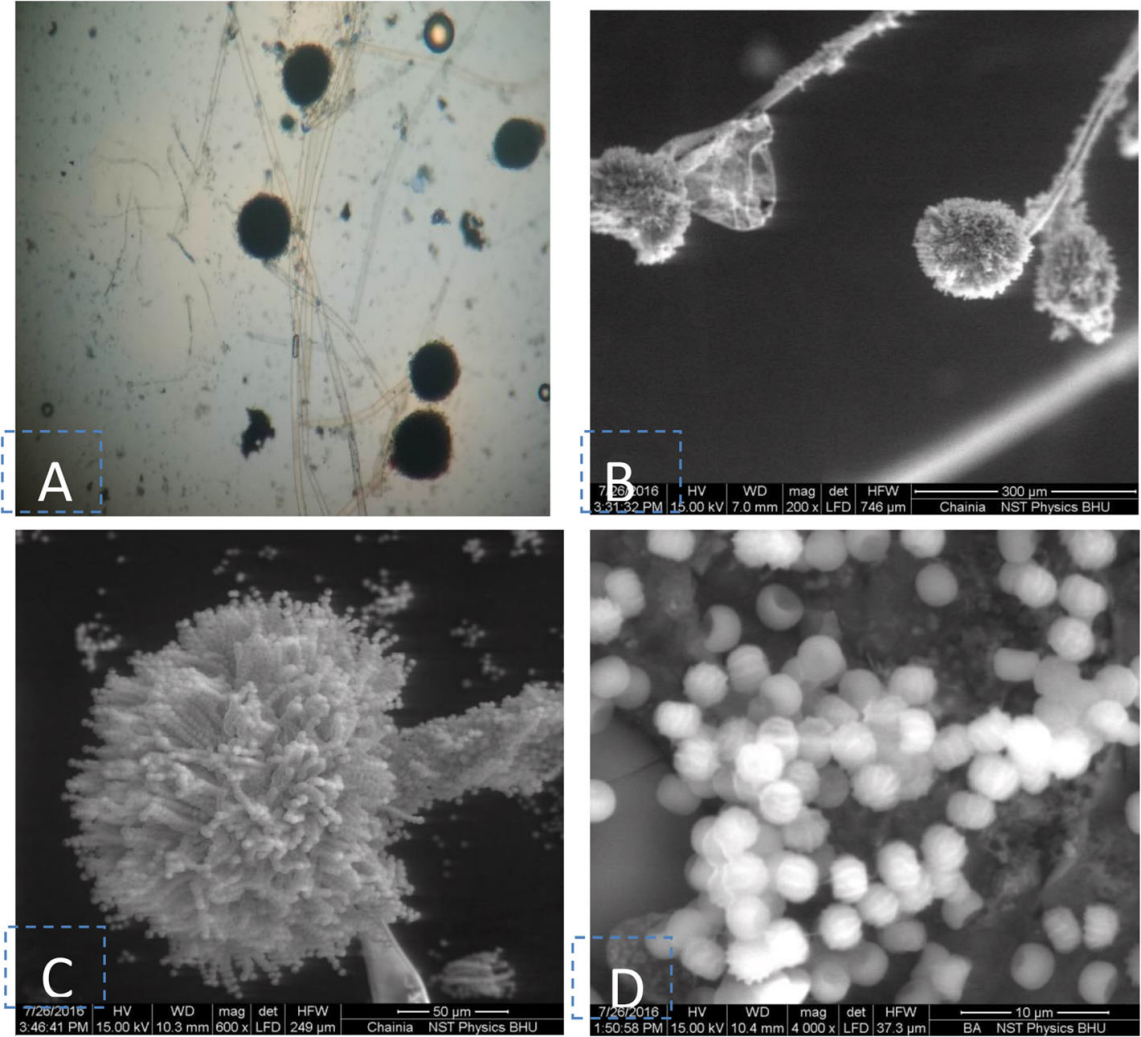
Spore chain morphology of Aspergillius sp.BN-2 , **a**-light microscope 10X;**b**- ESEM (200X); **c**-500X; **d**-spore surface (4000 X)

**Fig. 6:**
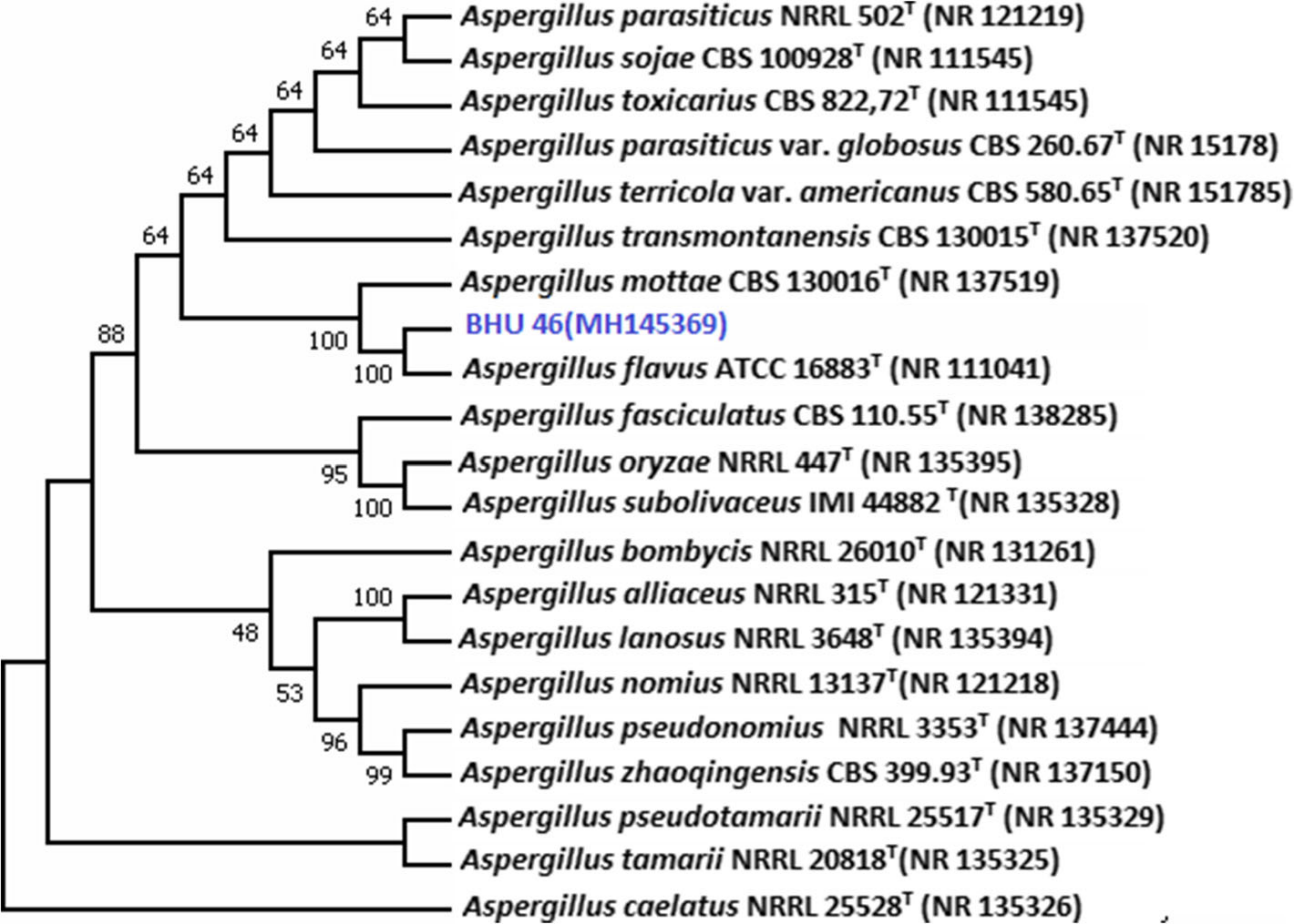
Phylogenetic tree of isolate BHU-46 obtained by maximum likelihood method based on the Kimura 2-parameter model. The percentage of replicate trees in which the associated taxa clustered together in the bootstrap test (1000 replicates) are shown next to the branches. The position of Isolate BHU 46 is shown with blue color. It is most closely related with *Aspergillus flavus* and supported by boot strap value of 100

**Fig. 7:**
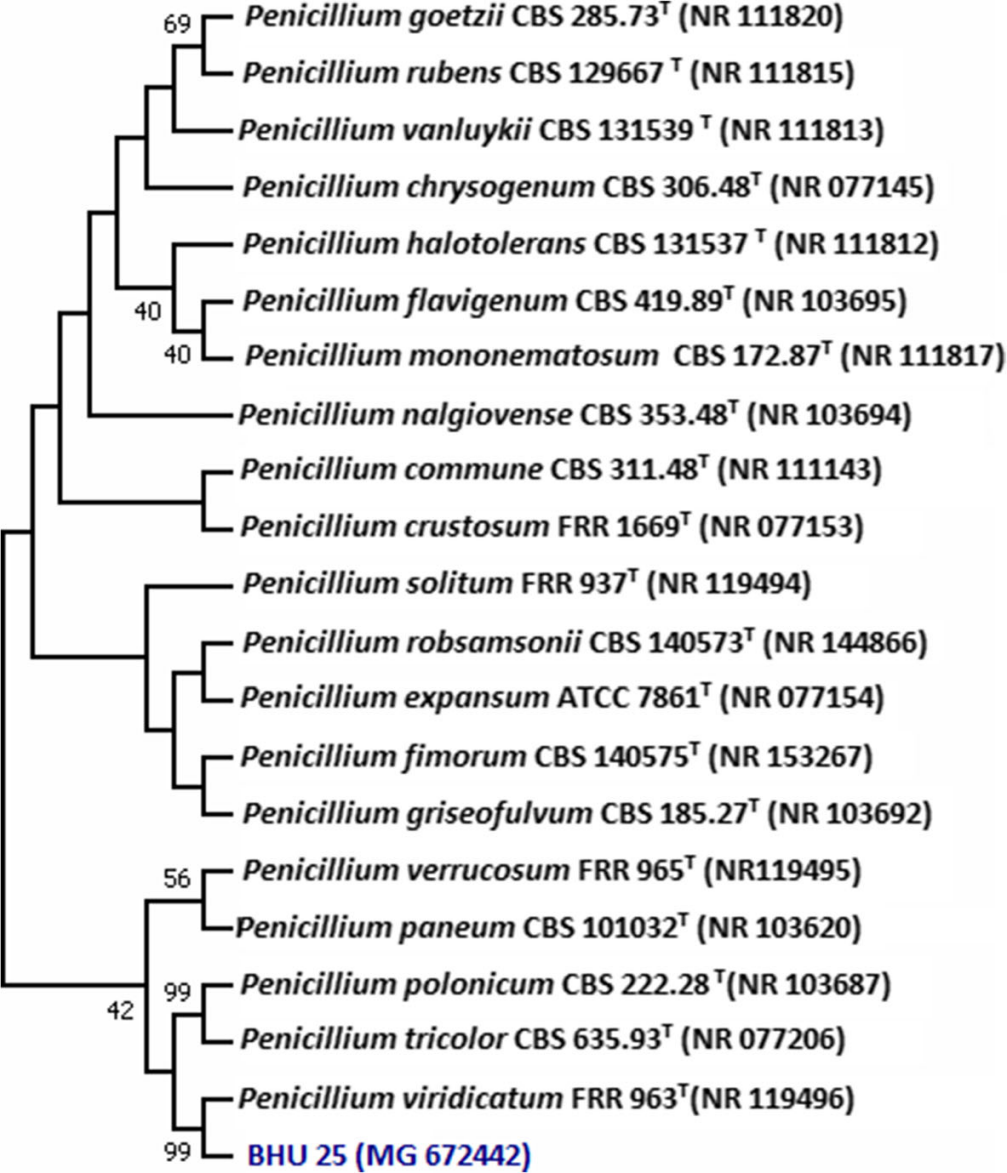
Phylogenetic tree of Isolate BHU-25 obtained by maximum likelihood method based on the Kimura 2-parameter model. The percentage of replicate trees in which the associated taxa clustered together in the bootstrap test (1000 replicates) are shown next to the branches. The position of Isolate BHU 46 is shown with blue color. It is most closely related with *Penicillium viridicatum* and supported by boot strap value of 99

Based on colony morphology and spore chain morphology isolates, BHU-25 and BHU-46 were identified as *Penicillium* sp. and *Aspergillus* species, respectively (Figs. 3 and 4). ESEM study showed that spore chains of *Penicillium* were composed of spores, all of which had smooth surfaces (Fig. 4) while in *Aspergillus* species, the chains were composed entirely of rugose surfaces (Fig. 5). ITS sequences were submitted to the NCBI Genbank, the USA with the accession number given in Table 3. The pairwise sequence similarity index with type strains of most promising isolates has been given in Table 3. The pairwise sequence analysis showed that the isolates BHU-25 and BHU-46 are most closely related with *Penicillium viridicatum* FRR963^T^ and *Aspergillus flavus* ATCC16883^T^ with a sequence similarity of 99.1% and 99.4%, respectively. The evolutionary history was inferred by using the maximum likelihood method based on the Kimura 2-parameter model revealed the position of BHU-46 with *Aspergillus flavus* ATCC16883^T^ with a confidence level of 100% while that of BHU-25 with *Penicillium viridicatum* FRR963^T^with confidence of 99% (Figs. 6 and 7).

**Table 3:** Genbank accession number of promising isolates

S.No.	Isolates	GenBank Accession numbers	Sequence identities (Diff./total nt)	Most closely related with
1	BHU-25	MG672442	99.1% (5/587)	*Penicillium viridicatum* FRR963^T^
2	BHU-46	MH145369	99.4% (518/521)	*Aspergillus flavus* ATCC16883^T^

## Discussion

Endophytic fungi have been explored from various plant sources for their industrial potential. Therefore, researchers focused on terrestrial plants for endophytic fungi. In this context, the present study plants the diversity of endophytic fungi associated with *Tradescantia pallida*, Zea *mays*, and *Trifolium alexandrinum* has been explored. *Aspergillus*, *Penicillium*, and *Ganoderma* species recovered highest from the stem of *Tradescantia palida*. *Fusarium* was dominant in the stem and leaf of *Zea mays*, *Penicillium*, *Aspergillus*, *Ganoderma*, *Cladosporium*, *Fusarium*, and *Alternaria* were recovered from the *Trifolium alexandrium.* This finding suggests that *Aspergillus* is most commonly associated with plants endophytes*.* These findings are similar to previous reports by other authors [24]. However, most dominating fungal endophytes belonging to the genus *Fusarium*, *Sarocladium*, *Aspergillus*, and *Penicillium* are not tissue-specific [25].

The diversity index is a quantitative indicator that measures the number of different species and the degree of distribution of individuals among those species. The Shannon diversity index (*H*) is commonly used to characterize species diversity in a community. The Shannon index in *Tradescantia pallida* is highest in leaves. These findings are similar to the results of Choudhary et al. [18], in which they also reported the highest Shannon index in leaves. While in *Zea mays* and *Trifolium alexandrinum*, it is highest in the stem which is comparable with previous studies [18, 26]. Simpson’s diversity index tells us about species dominance. It considers both the number of species present and their relative abundance. Simpson’s diversity index (species dominance) is a measure of diversity that takes into account the number of species present, as well as the relative abundance of each species. The diversity of species increases as the richness and evenness increase. Its value (D) ranges between 0 and 1, where 1 represents infinite diversity and 0 represents no diversity. The Simpson’s index was highest in the case of *Zea mays* while Menhinick’s index was highest in roots of *Trifolium alexandrinum.* Species riches are indicated by Menhinick’s index. As per our knowledge, no comparative data is available on the endophytic diversity of the above plants taken for the study. When we compared the diversity with other plants the results are comparable. Li et al. [27] reported that in the root, the fungal richness was significantly higher in *Salsola nitraria* other plants. Furthermore, the fungal richness was significantly higher in roots than in stems. Moreover, in recent times, the diversity analysis of fungal endophytes has been performed by several authors and revealed the discovery of new species producing novel metabolites. Diversity analysis is also helpful in understanding the role of endophytes in ecosystems [28, 29].

Among the various microbial enzymes available, starch processing enzymes are one of the prominent groups applied in processes like brewing, baking, and pharmaceuticals. Amylases are the group of enzymes that are generally used for the processing of starch [30, 31]. The starch processing enzymes are classified into four different categories which include exoamylases, endoamylase, transferases, and debranching enzymes. Among the different hydrolyzing enzymes, the α-Amylases and pullulanase are the more versatile enzymes used in the industrial sector and their contribution is about 25% of the whole enzyme market [32]. These enzymes act randomly on starches, glycogen, and oligosaccharides to yield-reducing sugar. Pullulanase is the significant industrial group in the 13 glycosyl hydrolases category, known as the α-amylases family [8, 9]. They hydrolyze the glycosidic bonds in the starch during the saccharification process and yield glucose, maltose, and maltotriose syrups. These products have found their significant applications in food and other related industries. Being a member of starch hydrolyzing enzymes, pullulanase hydrolyzes both α-1,6 and α-1,4 bond in pullulan and on other carbohydrates [33–35]. Therefore, the endophytes were screened for their pullulanase and amylase-producing capabilities. The recovery of pullulanase-producing endophytic fungi was very less. But the importance of pullulanase is high as compared to amylase because it has both α-1,6 and α-1,4 hydrolyzing ability. In SSF using rice bran (agro-waste) as substrate, a good yield was recovered and merit future interest for scale up the process.

Based on colony morphology, spore chain morphology, and ITS sequence analysis, the isolates BHU-25 and BHU-46 were identified as *Penicillium* sp. and *Aspergillus* species, respectively. Waqas et al. [36] reported *Penicillium* and *Aspergillus* species from tissues of sunflower (*Helianthus annuus* L.). Endophytic fungi like *Synnematous* sp., *Nodilusporium* sp., and *Acremonium* sp. have reported starch degrading enzymes [37, 38] reported forty-four endophytic fungal strains belonging to genus *Penicillium*, *Cladosporium*, *Monodictys*, *Phoma*, *Tetraploa*, and *Acremonium*, producing enzymes of industrial importance. However, pullulanase-producing endophytic fungi have not been reported so far.

## Conclusions

*Tradescantia pallida*, Zea *mays*, and *Trifolium alexandrinum* are rich sources of endophytic fungi*.* The Shannon index in *Tradescantia pallida* was highest in leaves while in *Zea mays and Trifolium alexandrinum*, it is highest in the stem. The Simpson’s index is highest in the case of *Zea mays* stem and root. Species richness was indicated by Menhinick’s index, and it was found that this value was highest in the roots of *Trifolium alexandrinum.* As per our knowledge, no comparative data is available on the endophytic diversity of the above plants taken for the study. The endophytes from these plants can produce pullulanase (2.38%) and amylase (7.94%). The recovery of pullulanase-producing endophytic fungi was very less. But the importance of pullulanase is high as compared to amylase because it has both α-1,6 and α-1,4 hydrolyzing ability. This is the first report of pullulanase from endophytic *Aspergillus* and *Penicillium* producing pullulanase.

## Supplementary Information

**Additional file 1.** The additional file contains Table-S1 showing preliminary screening for polysaccharides hydrolyzing activity. It also contains Fig. S1 which shows the occurrence of amylase and pullulanase-producing endophytes. Table S1 shows major isolates producing amylase and Pullulanase. Three isolates BHU-20, BHU25, and BHU46 showed both amylase and pullulanase activity. Isolates BHU-25 and BHU-46 showed higher activity in Preliminary screening hence taken for further studies. Fig. S1 shows that amylase-producing isolates were dominant as compared to pullulanase-producing fungi.

## Data Availability

All data generated or analyzed during this study are included in this article.

## Abbreviations

ITS: Internal transcribed spacer
SSF: Solid-state fermentation
DNA: Deoxyribonucleic acid
PCR: Polymerase chain reaction
MgCl_2_: Magnesium chloride
dNTP: Deoxynucleotide triphosphates
SSF: Solid-state fermentation
UV: Ultraviolet
ESEM: Environmental scanning electron microscope
NCBI: National Centre for Biotechnology Information
