# Supplementary material for: Exploring the diversity of endophytic fungi and screening for their pullulanase-producing capabilities
Source: J Genet Eng Biotechnol. 2021 Jul 29;19:110. doi: 10.1186/s43141-021-00208-0 (PMC8322383; doi:10.1186/s43141-021-00208-0)
Supplement: Supplementary file 1 — Additional file 1. The additional file contains Table-S1 showing preliminary screening for polysaccharides hydrolyzing activity. It also contains Fig. S1 which shows the occurrence of amylase and pullulanase-producing endophytes. Table S1 shows major isolates producing amylase and Pullulanase. Three isolates BHU-20, BHU25, and BHU46 showed both amylase and pullulanase activity. Isolates BHU-25 and BHU-46 showed higher activity in Preliminary screening hence taken for further studies. Fig. S1 shows that amylase-producing isolates were dominant as compared to pullulanase-producing fungi. [file 43141_2021_208_MOESM1_ESM.docx]

**Additional file 1**

**Table-S1:** Preliminary screening for polysaccharides hydrolyzing activity

| **S.No.** | **Sample** | **Genera** | **Starch** | **Pullulan** |
| --- | --- | --- | --- | --- |
| 1 | BHU-15 | *Aspergillus* sp. | ++ | - |
| 2 | **BHU-20** | ***Ganoderma* sp.** | **++** | **++** |
| 3 | **BHU-25** | ***Penicillium* sp.** | **++** | **+++** |
| 4 | **BHU-46** | ***Aspergillus* sp.** | **++** | **+++** |
| 5 | BHU-60 | *Fusarium* sp. | ++ | - |
| 6 | BHU-67 | *Cladosporium* sp. | ++ | - |
| 7 | BHU-81 | *Aspergillus* sp. | ++ | - |
| 8 | BHU-101 | *Aspergillus* sp. | ++ | - |
| 9 | BHU-103 | *Fusarium* sp. | ++ | - |
| 10 | BHU-112 | *Ganoderma* sp. | ++ | - |

Symbols used: +, week; ++, Good; +++, Excellent; - No hydrolysis

#
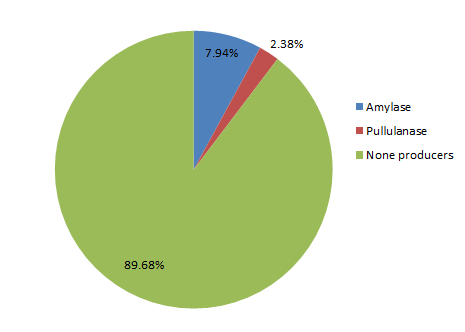


**Fig. S1.** Occurrence of amylase and pullulanase producing endophytes
